# Progress in the Cross-Organ Biomarker oxLDL in Promoting Pathological Neovascular Diseases

**DOI:** 10.3390/antiox15020182

**Published:** 2026-02-01

**Authors:** Yuekai Wu, Xinyi Lao, Xiaoling Su, Haoren Chen, Changzhen Fu, Qingping Liu

**Affiliations:** 1 Joint Shantou International Eye Center of Shantou University and the Chinese University of Hong Kong, Shantou 515041, China; 2 Medical College, Shantou University Shantou 515041, China

**Keywords:** neovascular diseases, oxidized low-density lipoprotein (oxLDL), lipid peroxidation products, chronic inflammation, cellular functional reprogramming, common mechanisms of angiogenesis

## Abstract

Neovascular diseases, such as neovascular ophthalmopathy, atherosclerosis, and tumors, are characterized by pathological angiogenesis, leading to the formation of leaky, tortuous, and immature blood vessels, often accompanied by chronic inflammation and tissue damage. Among the multiple drivers of angiogenesis in these conditions, the role of oxidized low-density lipoprotein (oxLDL) has garnered increasing attention. Formed from low-density lipoprotein (LDL) under oxidative stress, oxLDL acts as a cross-organ biomarker that systemically impacts multiple organs via the circulatory system, exerting a pivotal pro-angiogenic effect. This review focuses on elucidating the common molecular mechanisms by which oxLDL and its downstream lipid peroxidation products accumulate in disease-specific microenvironments. This accumulation activates inflammatory and oxidative stress pathways in macrophages and endothelial cells, modulating their functional reprogramming and thereby driving pathological neovascularization. Our aim is to provide an integrated framework for understanding the complex role of oxLDL as a cross-organ biomarker in multisystem neovascular diseases and to offer a theoretical basis for its potential as a therapeutic target.

## 1. Introduction

Neovascular diseases, a group of conditions encompassing neovascular ophthalmopathy, atherosclerosis, and tumors, share a core pathological feature: abnormal angiogenesis [1], a process frequently accompanied by chronic inflammation and tissue damage. Although therapies targeting vascular endothelial growth factor (VEGF) have become the mainstream clinical intervention strategy, issues such as therapeutic resistance and disease recurrence in some patients underscore the complex heterogeneity of neovascular pathogenesis and the limitations of clinical treatments. Consequently, exploring key upstream pathogenic factors has emerged as a critical research focus in recent years [2,3,4,5,6,7,8,9]. Among these upstream drivers, oxidized low-density lipoprotein (oxLDL), generated through systemic oxidative stress and lipid metabolism dysregulation, is increasingly recognized as a pivotal pathogenic molecule [10,11,12,13,14,15,16,17,18,19].

Circulating throughout the body, oxLDL can systemically affect multiple organs via the circulatory system. Through common mechanisms such as activating inflammation and amplifying oxidative stress, oxLDL ultimately leads to the dysregulation of the VEGF signaling pathway, thereby driving the development and progression of various vascular diseases, including neovascular ophthalmopathy [20], atherosclerosis [21], and tumors [22]. This cross-organ angiogenic effect provides a strong rationale for targeting oxLDL in therapy [23].In recent years, research has expanded beyond the overall effects of oxLDL to its diverse downstream lipid peroxidation products. These include highly reactive aldehydes [24], hydroxy fatty acids [25,26], oxidized phospholipids (oxPL) [27,28], and oxysterols [29,30], which possess distinct biological functions, providing a novel and more refined molecular perspective on the pro-angiogenic mechanisms of oxLDL.

This review aims to provide an updated summary of recent advances concerning oxLDL and its downstream lipid peroxidation products as cross-organ biomarkers. We will focus on elucidating their pathogenic roles in neovascular ophthalmopathy, atherosclerosis, and tumors. Based on this analysis, we will distill the common molecular mechanisms that drive cross-organ angiogenesis and, finally, explore the potential of oxLDL as a unifying therapeutic target.

While the pathological roles of oxLDL and its downstream lipid peroxidation products in specific diseases such as atherosclerosis, tumors, and neovascular ophthalmopathy are widely recognized, a cross-organ perspective unifying their actions across these distinct pathological microenvironments remains lacking. Therefore, this review aims to integrate recent advances to position oxLDL and its metabolites as central cross-organ biomarkers. To ensure a thorough analysis, we identified relevant literature through a structured search of the PubMed and Web of Science databases up to December 2025, using keywords including ‘oxLDL’, ‘lipid peroxidation’, ‘neovascularization’, and ‘angiogenesis’ within the contexts of ophthalmopathy, atherosclerosis, and tumors. We focus on elucidating their pathogenic roles in these diverse conditions, distilling the common molecular mechanisms that drive pathological angiogenesis, and finally, exploring the potential of oxLDL as a unifying therapeutic target. By qualitatively synthesizing current evidence, this review proposes a novel ‘cross-organ’ framework. Analogous to the concept of ‘pan-cancer’ analysis, we use this term to describe the conserved pathogenic mechanisms of oxLDL that transcend anatomical boundaries. Our goal is to deepen the understanding of shared pathophysiology in vascular-related diseases.

## 2. Generation of oxLDL and Its Downstream Lipid Metabolites

### 2.1. Generation of oxLDL

oxLDL is the product of the oxidative modification of LDL. LDL, the primary carrier of cholesterol in the human body, is a lipoprotein particle composed of apolipoprotein B-100 (apoB-100) and various lipid molecules. Its core lipid components are cholesteryl esters (CE), triglycerides (TG) and phospholipids, while its surface is predominantly composed of free cholesterol (FC) and phospholipids (PL), mainly phosphatidylcholine (PC) and sphingomyelin (SM). Additionally, LDL particles contain lysophosphatidylcholine (Lyso-PC), esterified and free polyunsaturated fatty acids (PUFAs), phosphatidylethanolamine (PE), diacylglycerol (DAG), ceramide (CER), and minor amounts of phosphatidylinositol. The conjugated double-bond systems, structural characteristics of the sterol rings, and electronic effects within these lipid constituents collectively form the key molecular basis for LDL’s susceptibility to oxidation [31,32].

**Figure 1:**
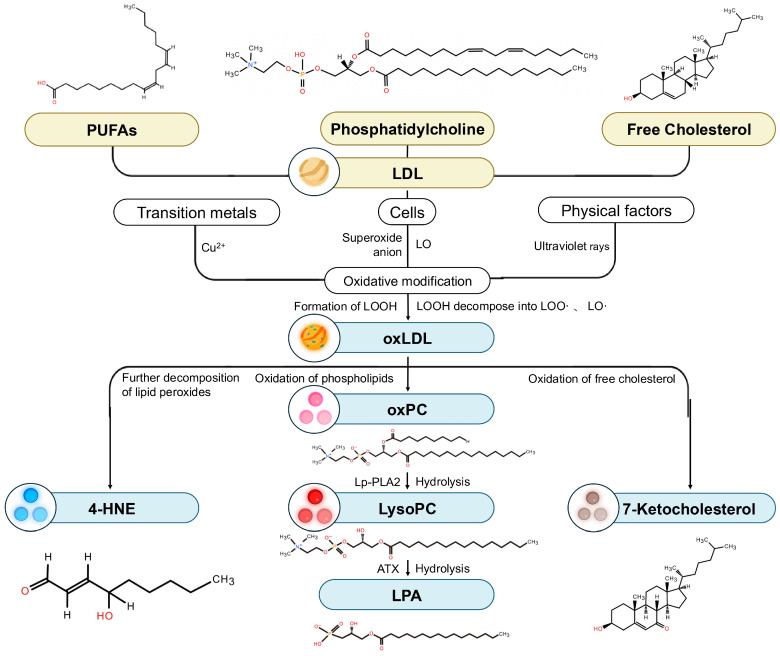
Formation of oxidized low-density lipoprotein (oxLDL) and its downstream lipid peroxidation products. Low-density lipoprotein (LDL) is primarily composed of lipid constituents including phosphatidylcholine, polyunsaturated fatty acids (PUFAs), and free cholesterol. Under various oxidative modifications mediated by cells, transition metals, or physical factors, LDL is converted into oxLDL. The main downstream products of oxLDL include: (1) highly reactive aldehydes, such as 4-hydroxynonenal (4-HNE); (2) oxidized phosphatidylcholines (oxPC), which can be further hydrolyzed by lipoprotein-associated phospholipase A2 (Lp-PLA2) to lysophosphatidylcholine (Lyso-PC), and subsequently by autotaxin (ATX) to lysophosphatidic acid (LPA); and (3) oxysterols, such as 7-ketocholesterol (7-KC). Created in BioRender. Wu, Y. (2026) https://BioRender.com/oya5e4n (accessed on 5 January 2026).

The oxidative modification of LDL is primarily initiated by free radicals in the body, such as oxygen and hydroxyl radicals, which convert lipids into lipid radicals and lipid peroxyl radicals, ultimately yielding lipid hydroperoxides (LOOH) [31]. This oxidative process is initiated by various cell types, including endothelial cells, macrophages, intimal smooth muscle cells, and monocytes, all of which can oxidatively modify LDL [31]. The precise mechanisms of cell-mediated LDL oxidation are still under investigation, but current perspectives suggest that the process is closely linked to cell-generated superoxide anions. Intracellular lipoxygenase (LO) also plays a critical role by catalyzing the incorporation of molecular oxygen into PUFA chains, thereby initiating the oxidative cascade [33]. These lipid oxidation products ultimately form covalent modifications with the apoB protein of LDL, altering its conformation and leading to the formation of oxLDL [30,34]. Furthermore, transition metals, such as Cu^2+^, can catalyze a self-propagating chain reaction of LOOH, generating a large quantity of peroxidation products [31]. Physical factors, including ultraviolet irradiation, can also induce oxidative modification of LDL [35] (Figure 1).

### 2.2. Generation and Biological Properties of Key Downstream Lipid Peroxidation Products of oxLDL

The downstream lipid metabolites of oxLDL include highly reactive aldehydes such as 4-hydroxynonenal (4-HNE) and malondialdehyde (MDA) [24]; hydroxy fatty acids like hydroxyoctadecadienoic acid (HODE) and hydroxyeicosatetraenoic acid (HETE) [25,26]; oxidized phospholipids, including oxidized phosphatidylcholines (oxPC) and Lyso-PC [27,28]; and oxysterols, such as 7-ketocholesterol (7-KC) and 24(S)-hydroxycholesterol (24-OHC) [29,30].

#### 2.2.1. Highly Reactive Aldehydes

4-HNE, a highly reactive aldehyde formed during the oxidation of oxLDL, is considered a reliable marker of oxidative stress [36]. LDL is rich in both free and esterified PUFAs, such as linoleic acid, γ-linolenic acid, and arachidonic acid. The oxidative modification of these molecules triggers the cleavage of fatty acid chains, culminating in the formation of highly reactive terminal functional groups, such as aldehydes. This process represents the primary pathway for 4-HNE formation [36,37].

The potent biotoxicity of 4-HNE stems from its unique α,β-unsaturated aldehyde chemical structure [38]. The aldehyde group and carbon-carbon double bond in this structure confer a high degree of electrophilicity, enabling it to readily form irreversible covalent bonds with cysteine, histidine, and lysine residues on proteins via Michael addition reactions and Schiff base formation [36,39]. This widespread formation of protein adducts, a process known as protein carbonylation, leads to the functional inactivation or aberrant activation of target proteins and directly triggers the dysregulation of key signaling pathways, including NF-κB and nuclear factor erythroid 2-re lated factor 2(Nrf2) [38,39]. Therefore, 4-HNE is not merely an end-product of oxidative damage but also a critical second messenger. It amplifies upstream lipid peroxidation into more persistent and widespread cellular dysfunction, inflammatory responses, and apoptosis, thereby playing a crucial role in driving the progression of neovascular diseases [36,38].

#### 2.2.2. Oxidized Phospholipids

Phosphatidylcholine is a major component of phospholipids. oxPC are generated from the oxidation of unsaturated fatty acids within the PC on the LDL surface. While the chain length is preserved, this oxidative modification introduces functional groups such as hydroxyl and hydroperoxyl moieties [37]. These structural alterations create damage-associated molecular patterns (DAMPs) that can be recognized by immune cells [37]. This unique molecular signature is efficiently bound by scavenger receptors, such as CD36, which triggers downstream inflammatory and immune responses, positioning oxPC as a key molecule linking lipid peroxidation to pathological signal transduction [37,40].

oxPC is hydrolyzed by plasma-type platelet-activating factor acetylhydrolase (plasma PAF-AH)—also known as lipoprotein-associated phospholipase A2 (Lp-PLA2)—to produce Lyso-PC. Within oxLDL, the amount of Lyso-PC can reach up to one-third of the total PC. It is the most abundant lysoglycerophospholipid in human blood [41] and is primarily generated through the hydrolysis of oxPC, a process specifically mediated by oxLDL-activated Lp-PLA2 [42] (Figure 1).

The unique chemical structure of Lyso-PC is the basis of its biological activity. Unlike PC molecules, which possess two hydrophobic fatty acid chains, Lyso-PC contains only a single fatty acid chain, resulting in a “cone-shaped” geometric conformation [43] and a detergent-like amphipathic property [44]. This structural feature allows it to easily insert into and perturb the lipid bilayer of cell membranes, disrupting membrane integrity and affecting the function of membrane proteins. This serves as the physicochemical basis for inducing various pathological effects, including endothelial damage and inflammation [44].

Indeed, this direct physical disruption of the cell membrane constitutes the critical initiating step in the diverse pro-atherogenic effects of Lyso-PC, such as inducing endothelial cell injury, activating inflammatory signals, and promoting immune cell chemotaxis. Furthermore, in various pathophysiological processes, Lyso-PC can be hydrolyzed by a secreted phospholipase D enzyme known as autotaxin (ATX) to generate a more potent lipid mediator: lysophosphatidic acid (LPA). As a critical signaling molecule, the resulting LPA activates downstream pathways and plays a pivotal role in pathological processes such as angiogenesis, inflammation, and therapeutic resistance [45,46,47,48].

#### 2.2.3. Oxysterols

7-KC, a principal oxysterol in oxLDL, is generated from the oxidation of FC and represents a highly abundant and toxic component, accounting for approximately 30% of the total sterols [49,50]. The key structural distinction between 7-KC and cholesterol is the presence of a ketone group (C=O) at the C7 position, a modification that significantly increases the molecule’s polarity and confers potent biological activity and cytotoxicity [51]. This altered polarity prevents 7-KC from integrating stably into the cell membrane like cholesterol. Instead, it embeds more superficially, severely disrupting the ordered structure of membrane microdomains known as “lipid rafts” [51,52]. The disruption of lipid raft architecture further amplifies reactive oxygen species(ROS)-mediated activation of NF-κB and caspase-dependent signaling pathways, which is a core mechanisms underlying the pronounced pro-inflammatory, pro-oxidative, and pro-apoptotic effects of 7-KC [51].

7-KC has been found to accumulate in large quantities in macrophages cultured in high-glucose environments and is highly enriched in the serum of patients with cardiovascular disease [53]. 7-KC exhibits significant pro-inflammatory and pro-oxidative effects in vivo, markedly impacting endothelial dysfunction, smooth muscle cell migration, and angiogenesis [29]. Transported via the bloodstream, 7-KC deposits in the vascular systems of multiple organs, including arterial walls, cerebral blood vessels, and the retina, demonstrating its cross-organ pathogenic properties as a major downstream lipid product of oxLDL [15].

## 3. Interaction of oxLDL and Its Downstream Lipid Products with Major Receptors

The complex biological effects of oxLDL and its downstream lipid peroxidation products are contingent upon their recognition and binding by specific cell surface receptors. These mainly include LOX-1, CD36, SR-A, and TLRs [54,55].

### 3.1. LOX-1

Lectin-like oxidized low-density lipoprotein receptor-1 (LOX-1) is a single-pass transmembrane glycoprotein belonging to the C-type lectin superfamily, which functions as a 52 kDa homodimer in humans [56]. LOX-1 plays a particularly critical role in endothelial cells, where its expression is regulated by multiple pathological factors [57]. It is also expressed in macrophages, vascular smooth muscle cells, and various tumor cells [58]. As a specific receptor for oxLDL, LOX-1 comprises four domains: a short N-terminal cytoplasmic domain (CD: 1–36 aa), a transmembrane domain (TM: 37–57 aa), a neck domain (NECK: 58–150 aa), and a C-type lectin-like extracellular C-terminal domain (CTLD: 151–273 aa) [59]. The CTLD specifically recognizes the modified apoB moiety of oxLDL particles, leading to the formation of a disulfide-linked, heart-shaped homodimer. This dimer can further assemble into larger functional oligomers through non-covalent interactions [58]. In endothelial cells, this process is a critical initiating step that triggers dysfunction and inflammatory responses, mediating not only lipid endocytosis but also serving as a crucial signal transduction node. Therefore, LOX-1-mediated signal transduction constitutes a critical link that directly translates the oxLDL stimulus into the pro-angiogenic functional reprogramming of cells [57,58].

### 3.2. CD36

CD36, a class B scavenger receptor, is a highly glycosylated 88 kDa integral membrane protein with two transmembrane helices [54]. CD36 is expressed on the surface of various innate and adaptive immune cells (such as macrophages and monocytes), as well as many non-immune cells, including platelets, certain specialized epithelial cells, and microvascular endothelial cells. Its expression and function are especially critical on macrophages, where it serves as a core receptor driving their transformation into foam cells [54]. Its large extracellular domain recognizes a variety of ligands. Notably, studies on macrophages have mapped a key oxLDL binding site to the amino acid region Met155-Arg183 on CD36. Within this, the crucial binding domain for oxLDL and its core component oxPC has been further pinpointed to the Leu157-Gln171 region, where two positively charged lysines, Lys164 and Lys166, are essential for the interaction [60]. This specific binding is a key step in activating the NOD-like receptor family pyrin domain containing 3(NLRP3) inflammasome and amplifying the inflammatory cascade [54,61]. Thus, CD36 is not merely an endocytic receptor for lipids but also a multifunctional signaling hub that couples lipid dysregulation with the pro-angiogenic chronic inflammatory microenvironment [54,61].

### 3.3. SR-A

Scavenger Receptor class A (SR-A) is a homotrimeric type II transmembrane glycoprotein with a monomeric molecular weight of approximately 77–80 kDa [55,62]. Key members of the SR-A family, including SCARA1 and MARCO, are highly expressed in macrophages [55]. Their core function is to mediate the endocytosis of oxLDL, driving the transformation of macrophages into foam cells and providing the material basis for the development of pathological neovascularization [62]. This process of foam cell formation creates a local hypoxic and inflammatory environment, a prerequisite for the subsequent initiation of new blood vessel growth [62].

### 3.4. TLRs

Toll-like Receptors (TLRs) are single-pass transmembrane proteins that play a central role in innate immune cells such as macrophages and are also expressed in non-immune cells like endothelial cells [63]. In the tumor microenvironment, they drive inflammation and tumor progression [64]. TLRs primarily recognize oxLDL and its derivatives as endogenous DAMPs [65]. For instance, oxLDL can activate TLR2 and TLR4 on the surface of human macrophages, inducing the secretion of pro-inflammatory cytokines such as IL-1β and IL-6, thereby triggering and exacerbating the inflammatory response [66,67,68]. Thus, the TLR pathway translates the presence of oxLDL into a potent pro-inflammatory signal, and this inflammatory response serves as a key driver of pathological neovascularization [64,66].

### 3.5. Major Receptors for Downstream Oxidized Lipids

In addition to the effects of the whole oxLDL particle, its key downstream lipid products also synergistically amplify the pathogenic effects of oxLDL by interacting with specific receptors. oxPC and LysoPC can be recognized by a multi-receptor network including CD36 and TLR4 [54,61,66,69], thereby triggering initial inflammatory signals [54,70,71]. The key oxysterol 7-KC can promote inflammatory responses by activating the TLR4 receptor [72] and upregulate CD36 expression to enhance macrophage phagocytosis of oxLDL [73]. The highly reactive aldehyde 4-HNE mainly forms adducts by modifying proteins [24,74]; for example, oxLDL containing HNE-apoB adducts can be recognized and taken up more efficiently by CD36 and SR-A, thus synergizing with oxLDL to exacerbate cell damage [24]. The binding of these products to their respective receptors collectively constitutes a more refined regulatory layer in the oxLDL pathogenic signaling network, providing diverse trigger pathways for subsequent inflammation and cellular dysfunction [24,54,72].

In summary, cells perceive and respond to oxLDL and its downstream lipid products through a multi-layered receptor system. Scavenger receptors such as LOX-1, CD36, and SR-A primarily mediate the binding, endocytosis, and accumulation of oxLDL and its various downstream lipid peroxidation products, providing the material basis for pathological changes like foam cell formation [54,58,62]. In contrast, TLRs function as signal sensors, recognizing specific lipid products as endogenous danger signals and activating innate immune responses [65].

## 4. The Cross-Organ Angiogenic Effects of oxLDL and Its Downstream Lipid Products in Neovascular Diseases

The recognition of oxLDL and its downstream lipid products by cell surface receptors constitutes the initiating step in a complex pathogenic cascade. These molecular interactions translate into tangible pathological damage.

### 4.1. Neovascular Eye Diseases

The ocular choriocapillaris possesses a fenestrated structure, which, during the acquisition of exogenous cholesterol, allows LDL to pass through and infiltrate Bruch’s membrane [75]; elevated oxLDL in the blood also enters Bruch’s membrane via this pathway [76]. The outer blood–retinal barrier, a component of the blood–ocular barrier formed by retinal pigment epithelium (RPE) cells, is characterized by high metabolic activity, constant light exposure, and active phagocytosis. Together, these features create a microenvironment of high oxidative stress, rendering retained LDL highly susceptible to oxidative modification [77,78,79]. Consequently, this locally generated oxLDL, together with oxLDL originating from the systemic circulation, constitutes the two primary sources of oxLDL in Bruch’s membrane.

RPE cells recognize and internalize oxLDL via the surface scavenger receptor CD36, a process that serves a clearance function under physiological conditions [76,80]. However, when lipid overload overwhelms this clearance capacity, CD36-mediated uptake shifts from a physiological protective role to one of pathological activation, leading to an imbalance between clearance and accumulation [80,81]. This shift results in the substantial retention and ectopic accumulation of oxLDL within Bruch’s membrane [76,78]. As the primary target of high local concentrations of oxLDL, RPE cells experience significant oxidative stress upon its accumulation. The intracellular accumulation of ROS is the key link between the oxidative stimulus and early RPE dysfunction [77,79,81]. Furthermore, excess oxLDL activates the NLRP3 inflammasome pathway via CD36 [80], inducing RPE cells to release large quantities of pro-inflammatory cytokines (e.g., IL-6, IL-8) and chemokines (e.g., Monocyte Chemoattractant Protein-1(MCP-1)) [77,79,80,81].

Various downstream lipid peroxidation products of oxLDL further amplify this pathological process. For instance, its key oxysterol product, 7-KC, can induce a state of senescence in RPE cells [82] and activate the senescence-associated secretory phenotype (SASP) through the mTOR-NF-κB signaling pathway, leading to the sustained secretion of high levels of pro-angiogenic factors such as VEGF-A [82].

Chemokines released by RPE cells effectively recruit circulating monocytes, which then differentiate into macrophages [79,81,83]. The extensive infiltration of macrophages into the subretinal space is a key pathogenic events in the formation of choroidal neovascularization (CNV) in neovascular age-related macular degeneration (nAMD) [79]. Critically, oxLDL-activated macrophages secrete signaling molecules such as Wnt3A, which in turn act on RPE cells to induce the production of large amounts of VEGF [84].

Stimulated by VEGF, choroidal endothelial cells undergo abnormal proliferation and migration, leading to the formation of pathological neovessels (i.e., CNV) [79,81,85]. These new vessels sprout from the choroid, grow upward, and penetrate Bruch’s membrane and RPE layer, which have been structurally compromised by inflammation and enzymatic degradation [86]. This process severely damages the integrity of the outer blood–retinal barrier; their tortuous morphology, immature structure, and abnormally high permeability collectively promote macular edema and hemorrhage, ultimately causing severe, vision-threatening complications [86].

**Figure 2:**
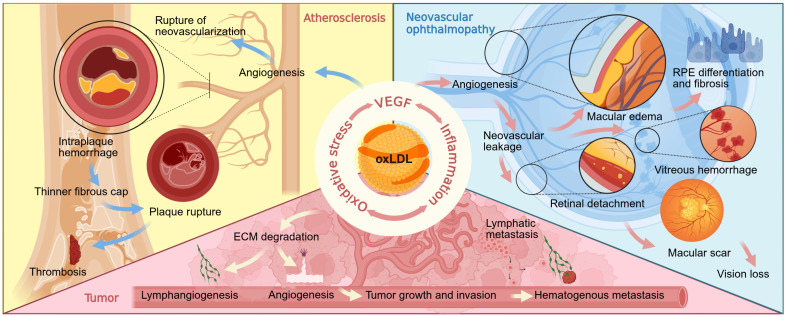
oxLDL as a cross-organ pathogenic factor in neovascular diseases. oxLDL drives multiple neovascular diseases by inducing oxidative stress and inflammation, which upregulates vascular endothelial growth factor (VEGF) expression. In ophthalmopathy, it triggers choroidal neovascularization, macular edema, and fibrosis, leading to vision loss. In atherosclerosis, it promotes intraplaque neovascularization, hemorrhage, and rupture. In tumors, it facilitates the generation of tumor blood and lymphatic vessels, supporting tumor growth, invasion, and metastasis. Created in BioRender. Wu, Y. (2026) https://BioRender.com/cikrxxp (accessed on 5 January 2026).

**Figure 3:**
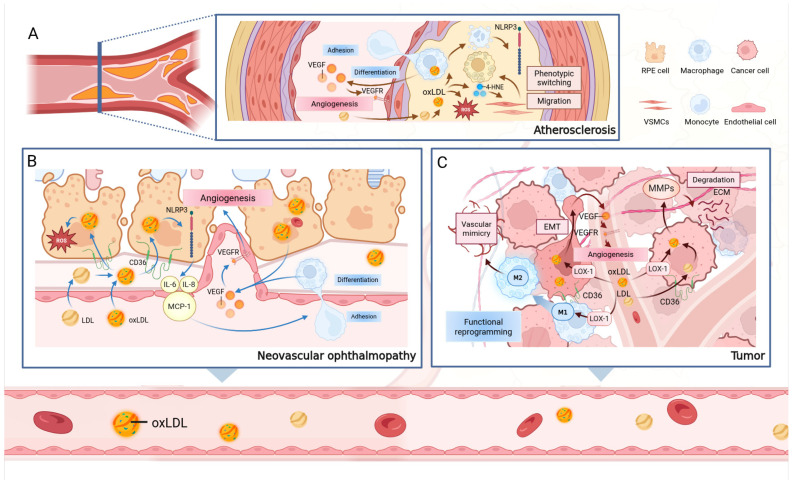
Disease-specific mechanisms of the oxLDL-mediated cross-organ angiogenic effect. The cross-organ angiogenic effect of oxLDL originates from its ability to activate macrophages and endothelial cells and drive neovascularization through interactions with specific cell types in different pathological microenvironments. (**A**) In atherosclerosis, oxLDL accumulates in the intimal layer of the arterial wall, promoting intraplaque neovascularization by recruiting macrophages, inducing foam cell formation, and driving the phenotypic remodeling of vascular smooth muscle cells (VSMCs). (**B**) In neovascular ophthalmopathy, oxLDL passes through the fenestrations of the choriocapillaris and deposits in Bruch’s membrane. Targeting retinal pigment epithelium (RPE) cells as a core component, it acts synergistically with macrophages to secrete VEGF, driving choroidal neovascularization. (**C**) In tumors, oxLDL accumulates in solid tumors, either through enrichment from circulation or via in situ oxidation of LDL, thereby inducing epithelial–mesenchymal transition (EMT) in tumor cells and M2 polarization of tumor-associated macrophages (TAMs) to promote the formation of a vascular network that supports tumor growth. Created in BioRender. Wu, Y. (2026) https://BioRender.com/cikrxxp (accessed on 5 January 2026).

The oxLDL-driven pathological process manifests in specific forms in different neovascular eye diseases due to variations in anatomical location and target cells. In nAMD, the core pathological process begins with the abnormal proliferation of CNV [87]. Persistent exudation and inflammation from these neovessels not only damage the retinal structure but also induce fibrosis of RPE cells [88]. This ultimately forms an irreversible macular scar that directly damages photoreceptor cells [89], leading to a permanent loss of central vision for the patient [87] (Figure 2). In proliferative diabetic retinopathy (PDR), the core pathology begins with retinal capillary damage and occlusion caused by chronic hyperglycemia, which leads to widespread retinal ischemia [90]. Unlike nAMD, the pathological damage in PDR primarily affects the “neurovascular unit” within the retina, especially Müller cells [90]. Studies have demonstrated that Müller cells can phagocytose lipid-rich hard exudates via the PPARγ-CD36 signaling axis, suggesting that oxLDL and its related products can also directly regulate Müller cell function in the PDR microenvironment through the CD36 receptor [91]. Under ischemic and inflammatory stimulation, activated Müller cells become the primary source of VEGF, directly driving the formation of fragile new blood vessels on the surface of the retina or optic disc [91]. These vessels often grow along the posterior hyaloid membrane as a scaffold [92,93] and are accompanied by the proliferation of fibrous tissue, gradually forming a contractile fibrovascular membrane [93]. The rupture of these pathological vessels can lead to vitreous hemorrhage, while the contraction of the fibrovascular membrane can cause tractional retinal detachment, both of which are major causes of severe visual impairment in patients [93,94] (Figure 3).

In summary, oxLDL drives the formation of pathological neovessels in the eye through a multi-cellular, synergistic cascade. This process begins with its abnormal accumulation at the basal aspect of RPE cells [76,78], hinges on the functional dysregulation of RPE cells [77,78,80,81,95], and, by recruiting and activating macrophages [79,81,83,84], creates a potent pro-inflammatory environment that activates endothelial cells [96], ultimately promoting pathological neovascularization (Figure 3B).

### 4.2. Atherosclerosis

In the systemic vascular disease of atherosclerosis, the pathogenic process of oxLDL and its downstream lipid peroxidation products begins with their infiltration across the vascular endothelial barrier and their subsequent retention and modification within the arterial wall. The deposition and retention of oxLDL in the arterial wall rapidly trigger a cascade of pathological effects, the core of which is the induction of chronic, sterile inflammation within the intima. The persistent lipid stimulus creates a potent pro-inflammatory environment, leading to the recruitment and infiltration of a large number of circulating monocytes into the arterial wall, where they differentiate into macrophages [97,98]. In the unique microenvironment of atherosclerosis, the physiological clearance function of macrophages becomes dysregulated due to lipid overload, culminating in their transformation into the iconic “foam cells” of the lesion [98,99,100]. As these foam cells undergo continuous apoptosis and necrosis, the large amounts of free cholesterol released from them form needle-shaped cholesterol crystals locally [101]. These crystals act as potent endogenous danger signals that perpetually activate the NLRP3 inflammasome, further amplifying the inflammatory response. This process becomes a key driver of plaque progression towards a complex phenotype [101] and also drives the complex phenotypic remodeling of vascular smooth muscle cells (VSMCs) [97]. VSMCs play a dual role in the progression of atherosclerosis. On one hand, they can differentiate into fibroblast-like cells that secrete extracellular matrix to form a protective “fibrous cap,” which is crucial for maintaining plaque stability [102,103]. However, oxLDL and its downstream lipid peroxidation products (such as 4-HNE) can disrupt this stabilizing function. They not only directly promote the proliferation and migration of VSMCs [104,105] but also induce their differentiation into unstable phenotypes, such as macrophage-like cells [106]. These differentiated cells participate in foam cell formation and, by releasing pro-inflammatory mediators, exacerbate local inflammation and promote the expansion of the hypoxic necrotic core [103,107]. Cutting-edge research has further revealed that oxLDL can induce a persistent pro-inflammatory phenotype in VSMCs through a mechanism of “trained innate immunity” [108]. Thus, by driving this pathological transformation of VSMCs, oxLDL acts in concert with inflammatory and hypoxic signals to create a potent pro-angiogenic microenvironment [102].

Building on this foundation, activated macrophages become the key effectors driving intraplaque neovascularization. In the inflammatory and hypoxic microenvironment shaped by oxLDL, the large population of infiltrating macrophages becomes a major source of angiogenic factors, including VEGF [109]. Concurrently, as the inflammatory response persists and the plaque volume increases, the plaque’s core gradually becomes a hypoxic necrotic core due to insufficient nutrient and oxygen supply [109,110]. This combination of hypoxic and inflammatory signals constitutes the most potent stimulus for inducing neovascularization, ultimately driving the formation of new blood vessels within the plaque [109].

These pathological new blood vessels, originating from the vasa vasorum, are primitive in structure, with incomplete and highly permeable walls that are prone to rupture. Their leakage and rupture introduce large quantities of red blood cells and lipids into the plaque, which dramatically expands the necrotic core, intensifies the inflammatory response, and weakens the structure of the fibrous cap. This leads to plaque instability and significantly increases the risk of plaque rupture and subsequent thrombosis, triggering severe clinical events such as myocardial infarction or stroke [111] (Figure 2).

In summary, oxLDL in atherosclerosis initiates a chronic inflammatory cascade centered on endothelial cell activation and macrophage foam cell formation, driven by its infiltration and accumulation in the arterial wall. It then drives VSMC phenotypic remodeling, fostering a pro-angiogenic microenvironment and leading to pathological intraplaque neovascularization. Therefore, oxLDL is not only an initiating factor in plaque formation but also a direct contributor to plaque vulnerability through the induction of pathological angiogenesis, serving as a key pathological link between lipid dysregulation and acute cardiovascular events (Figure 3A).

### 4.3. Tumors

In contrast to ocular and atherosclerotic microenvironments, the pathogenic role of oxLDL in tumors originates not only from the enrichment of circulating oxLDL but also from the retention and in situ oxidation of LDL within solid tumors. Studies have found that various tumor cells (e.g., breast and prostate cancer) actively upregulate the expression of oxLDL receptors LOX-1 and CD36 [112,113,114], mediating the capture and internalization of oxLDL. Furthermore, the vascular network of solid tumors is highly permeable, leading to an “Enhanced Permeability and Retention (EPR) effect,” which facilitates the passage of LDL nanoparticles across vessel walls and their prolonged retention within tumor tissue [115,116]. Subsequently, in the oxidative stress microenvironment induced by local hypoxia, these LDL particles are oxidized into oxLDL, ultimately leading to its significant accumulation in tumor tissue [117].

Studies have shown that oxLDL can induce tumor cells to secrete high levels of matrix metalloproteinases (MMPs), which degrade the extracellular matrix and facilitate endothelial cell migration and invasion, thereby promoting tumor angiogenesis [114]. oxLDL is also an effective inducer of epithelial–mesenchymal transition (EMT) in tumor cells, which not only enhances their invasive and metastatic capabilities but also prompts them to secrete more pro-angiogenic factors, amplifying the angiogenic signal [114,117]. Additionally, downstream products of oxLDL, such as lysophosphatidic acid (LPA), not only promote angiogenesis but also induce lymphangiogenesis within the tumor microenvironment, providing a crucial pathway for tumor metastasis via the lymphatic system [118,119,120] (Figure 2).

Within the tumor microenvironment (TME), the abundant accumulation of oxLDL does not only act directly on tumor cells but also initiate a tumor-specific neovascularization program by reprogramming tumor-associated macrophages (TAMs). In head and neck squamous cell carcinoma, the key oxLDL receptor LOX-1 is significantly upregulated on TAMs. Its high expression is closely associated with the M2 polarization of TAMs and correlates with poor clinical prognosis, suggesting that oxLDL is a key factor driving the pro-tumoral functional polarization of TAMs [121]. It has been demonstrated that polarized TAMs can induce and promote “vascular mimicry” (VM). Studies show that M2-type TAMs can significantly enhance the ability of glioblastoma cells to form VM networks [122]. Therefore, by inducing the M2 polarization of TAMs, oxLDL promotes a complex neovascularization mechanism distinct from traditional angiogenesis, providing an alternative blood supply route for the sustained growth and invasion of tumors.

In conclusion, within the TME, oxLDL, through its specific infiltration and in situ oxidative enrichment, initiates a pro-angiogenic cascade centered on the functional reprogramming of tumor cells and the polarization of TAMs. This ultimately leads to the formation of a structurally disorganized and functionally abnormal tumor neovasculature. Through this mechanism, oxLDL, by promoting angiogenesis, provides the necessary nutritional support for sustained tumor growth, invasion, and distant metastasis, highlighting its key regulatory role in malignant tumor progression (Figure 3C).

## 5. Common Molecular Mechanisms of oxLDL-Driven Neovascularization

The consistent pro-angiogenic effect of oxLDL across neovascular ophthalmopathy, atherosclerosis, and tumors suggests the existence of common underlying molecular mechanisms. This “cross-organ angiogenic” effect is not an isolated phenomenon but is rooted in a highly conserved pathophysiological network regulated by oxLDL. Its core cellular components include macrophages and vascular endothelial cells. Within these cells, oxLDL and its downstream lipid peroxidation products synergistically activate inflammatory responses, exacerbate oxidative stress, and induce cellular functional reprogramming. Through signal integration, multiple upstream signaling pathways are converged onto a VEGF-centric pro-angiogenic axis, providing an integrated molecular framework for understanding the intrinsic logic of oxLDL as a “cross-organ biomarker.”

### 5.1. Regulation of Macrophage Function by oxLDL

In the pathological microenvironments of various neovascular diseases, the recognition and internalization of oxLDL by macrophages through scavenger receptors such as CD36, LOX-1, and SR-A represents the critical initiating step of the subsequent pathological cascade [99,100]. oxLDL itself is a potent non-hypoxic activator of HIF-1α and can directly act on macrophages to strongly induce HIF-1α-dependent VEGF secretion [123]. Concurrently, the continuous uptake of oxLDL leads to substantial lipid accumulation within macrophages, transforming them into “foam cells” [99]. This process marks the initiation of a chronic inflammatory response, creating a pro-angiogenic inflammatory microenvironment. oxLDL also activates the canonical NF-κB signaling pathway by binding to cell surface receptors [124]. More in-depth studies have unveiled a “dual-signal model” for its activation of the NLRP3 inflammasome: first, oxLDL, via the CD36 receptor in coordination with Toll-like receptor 4/6 (TLR4/6), activates the NF-κB pathway to “prime” the transcription of *NLRP3* inflammasome components. Subsequently, the continuously endocytosed oxLDL forms cholesterol crystals within lysosomes, which in turn activates the NLRP3 inflammasome, ultimately promoting the maturation and release of the potent pro-inflammatory cytokines IL-1β and IL-18 [125].

The diverse downstream lipid products of oxLDL further fine-tune and amplify this inflammatory cascade. Its key pro-inflammatory component, LysoPC, not only induces the secretion of TNF-α and IL-6 through Protein Kinase C (PKC) and intracellular calcium signaling-dependent NF-κB pathways [126,127] but also prompts macrophages to release ATP via pannexin-1 channels. This ATP then acts as a “danger signal,” activating purinergic receptors on the cell surface and thereby triggering caspase-1-dependent maturation and release of IL-1β [128]. Similarly, the downstream product oxPC activates inflammatory responses through the canonical NF-κB signaling pathway; in macrophages, it engages the surface receptor TLR4 and, dependent on the downstream adaptor protein MyD88, activates the NF-κB pathway, leading to the production and secretion of IL-6 [70].Recent studies have revealed that key components of oxLDL, the oxidized phospholipids (oxPLs), can engage a non-canonical inflammasome activation pathway that is independent of classical potassium efflux and does not induce pyroptosis, yet allows for the sustained release of IL-1β, providing a novel mechanism for the maintenance of chronic inflammation [129,130,131,132].

This inflammatory cascade is further amplified by the key downstream products 7-KC and 4-HNE. 7-KC can significantly induce the expression of various inflammatory and angiogenic factors (including IL-6, IL-8, and VEGF) by activating the ERK1/2 and NF-κB signaling pathways [133,134], and it upregulates the CD36 receptor through a G-protein-coupled signaling cascade, forming a positive feedback loop of “inflammation-lipid uptake” [73]. Meanwhile, 4-HNE not only directly triggers NF-κB signaling via TLR4 [24,135] but also activates the intracellular cGAS-STING innate immunity pathway. This in turn activates TANK-binding kinase 1 (TBK1) and interferon regulatory factor 3 (IRF3), potently inducing the polarization of pro-inflammatory macrophages and exacerbating the inflammatory response [135].

The oxLDL-initiated inflammatory cascade is further amplified and sustained by another core pathological event it induces: oxidative stress. The accumulation of oxLDL itself exacerbates the local oxidative state, leading to the excessive production of ROS [133,136]. Numerous studies have confirmed that oxLDL is a potent inducer of oxidative stress in macrophages [137,138,139], a mechanism rooted in mitochondrial dysfunction. oxLDL disrupts mitochondrial homeostasis, inducing mitochondrial fission and leading to the massive generation of mitochondria-derived ROS (mito-ROS) [140,141]. Critically, this overproduced mito-ROS is not only a direct effector of cellular oxidative damage but also a key upstream signal that can effectively activate the NLRP3 inflammasome. This links oxidative stress and inflammation in a self-reinforcing vicious cycle [137].

**Figure 4:**
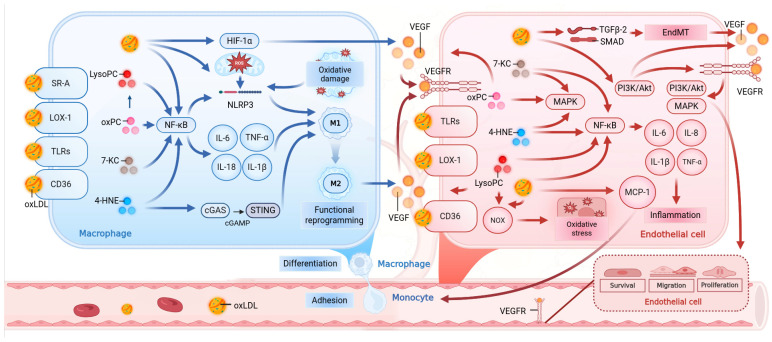
Cellular and molecular mechanisms of oxLDL-driven neovascularization in multiple cell types. In the microenvironment of neovascular diseases, oxLDL and its downstream lipid peroxidation products interact with macrophages and endothelial cells to activate a series of pro-angiogenic signaling pathways. Key mechanisms include: inflammatory responses mediated by scavenger receptors (e.g., CD36, LOX-1, and SR-A), such as the activation of NF-κB, NLRP3, and cGAS-STING pathways, which upregulate pro-inflammatory cytokines (e.g., TNF-α, IL-6); induction of oxidative stress and cellular functional reprogramming (e.g., polarization, EndMT); and the ultimate promotion of the expression of key pro-angiogenic factors like VEGF. The synergistic action of these multi-cellular and multi-pathway events collectively drives the formation of pathological neovessels. Created in BioRender. Wu, Y. (2026) https://BioRender.com/evkzmhp (accessed on 5 January 2026).

Ultimately, within the pathological microenvironment shaped by both inflammation and oxidative stress, macrophage function is reprogrammed, transforming them from immune surveillance cells into key effector cells that drive pathological neovascularization. In the early stages of disease, strong inflammatory signals primarily drive macrophage polarization towards the classic M1 phenotype, exacerbating local tissue damage [142]. However, as the disease progresses and the microenvironment changes, particularly under the influence of factors such as hypoxia, macrophages gradually transition towards the M2 phenotype, which promotes angiogenesis and tissue remodeling [143,144]. These reprogrammed M2-type macrophages can secrete large amounts of VEGF, becoming one of the key cellular sources driving the formation of pathological neovessels [145]. Therefore, this series of cascade events—initiating inflammation, amplifying signals via oxidative stress, and ultimately driving macrophage polarization toward a pro-angiogenic phenotype—constitutes the core common mechanism through which oxLDL exerts its “cross-organ angiogenic” effect in a multitude of neovascular diseases (Figure 4).

### 5.2. Regulation of Endothelial Cell Function by oxLDL

As the constituent units of the vascular barrier and direct participants in angiogenesis, vascular endothelial cells are one of the core targets of oxLDL. By inducing inflammatory activation, oxidative stress, and functional reprogramming in endothelial cells, oxLDL and its downstream lipid peroxidation products collaboratively establish a potent pro-angiogenic microenvironment. This process mirrors the regulatory mechanisms in macrophages, constituting the other key axis of its cross-organ pathogenic effects.

In the initial stages of neovascular diseases, oxLDL first enhances the capacity of endothelial cells to capture circulating immune cells by upregulating surface adhesion molecules, such as Vascular Cell Adhesion Molecule-1 (VCAM-1) and Intercellular Adhesion Molecule-1 (ICAM-1) [96]. Concurrently, oxLDL can activate the canonical NF-κB signaling pathway through various receptors (e.g., LOX-1 and TLR4) [146], promoting the robust secretion of chemokines like MCP-1 [147,148]. This, in turn, actively recruits monocytes to migrate, adhere, and infiltrate the arterial intima, laying the foundation for the establishment of a local inflammatory response [149].

Multiple downstream lipid peroxidation products of oxLDL synergistically amplify this initial inflammatory signal through parallel pathways. For instance, Lyso-PC can promote the secretion of inflammatory factors IL-6 and IL-8 by activating the TLR4/NF-κB signaling pathway [71] or by inducing endoplasmic reticulum stress [150]. Its metabolite, LPA, directly enhances the transendothelial migration of monocytes and stimulates endothelial cells to secrete additional chemokines and adhesion molecules [151,152,153]. Similarly, oxPC is primarily recognized by the scavenger receptor CD36 [69,154], activating the downstream p38 MAPK signaling pathway [155], thereby inducing the secretion of inflammatory chemokines (such as CXCL8/IL-8) and cytokines [154,155]. 7-KC mainly activates inflammatory pathways, including MAPK and NF-κB, via TLR4 [72], promoting the upregulation of mRNA expression for multiple pro-inflammatory cytokines, such as *IL-1β, IL-6, IL-8, TNF-α*, and the inflammatory mediator COX-2 [72,156,157], while also inducing ICAM-1 expression. In contrast, 4-HNE acts independently of specific receptors; through its high chemical reactivity, it directly enters the cell to activate MAPK and NF-κB pathways [74]. While promoting the secretion of inflammatory factors, it further upregulates the expression of adhesion molecules like ICAM-1, a central step in leukocyte-endothelial interactions during inflammation, ultimately causing and exacerbating the inflammatory state of the vascular endothelium [158].

This sustained inflammatory state, intertwined with the intense oxidative stress induced by oxLDL, collectively leads to endothelial cell dysfunction. The accumulation of oxLDL and its downstream lipid peroxidation products disrupts intracellular redox homeostasis, resulting in the massive generation of ROS [159,160]. The mechanisms are diverse, including the upregulation of NADPH oxidase (NOX) expression via LOX-1 [34,161], the uncoupling of endothelial Nitric Oxide Synthase (eNOS) [162], and the effects of its downstream products, such as the direct depletion of glutathione (GSH) by 4-HNE [163] and the activation of NOX5 by Lyso-PC [27]. Together, these pathways cause ROS production to far exceed the cell’s clearance capacity, thereby intensifying oxidative stress. This, in turn, inflicts multifaceted damage on endothelial cells, not only inducing cellular senescence and the SASP [159,160,164] but also directly triggering apoptotic programs [165], which ultimately compromises the integrity of the endothelial barrier [166].

Critically, this endothelial dysfunction, driven by the combined forces of inflammation and oxidative stress, can lead to a key functional reprogramming of endothelial cells, transforming them from barrier-maintaining cells into active pro-angiogenic cells. oxLDL can directly regulate the proliferation, migration, and phenotypic plasticity of endothelial cells. One of its classic mechanisms is the activation of PI3K/AKT and MAPK signaling pathways via the LOX-1 receptor, which in turn upregulates the expression of VEGF-A, its receptor VEGFR-2, and MMP-2 and MMP-9 [167,168]. Concurrently, the key downstream lipid peroxidation product of oxLDL, oxPC, can also synergistically upregulate VEGF expression through an autocrine mechanism, further amplifying this pro-angiogenic effect [169]. Moreover, oxLDL can induce Endothelial-Mesenchymal Transition (EndMT) by activating the TGF-β2/Smad signaling axis, causing endothelial cells to acquire mesenchymal characteristics and thereby greatly enhancing their migratory capacity and VEGF secretion [20,170].

The VEGF produced by oxLDL induction acts on endothelial cells in an autocrine or paracrine manner, binding to its specific receptor, VEGFR-2. This triggers receptor dimerization and autophosphorylation, which in turn activate multiple key downstream signaling pathways, such as the PI3K/Akt and MAPK pathways [167,171]. This signaling cascade, by regulating cell cycle progression and cytoskeletal rearrangement, significantly enhances the proliferative and migratory capabilities of endothelial cells [172], constituting the core driving force for neovascularization. This series of events clearly demonstrates that oxLDL integrates inflammatory and oxidative stress signals to ultimately reprogram endothelial cells into the key executive units of angiogenesis, thereby driving pathological neovascularization.

Notably, the neovascular network driven by oxLDL extends beyond the VEGF axis, orchestrating a multi-channel signaling network. First, oxLDL modulates vascular remodeling via the Angiopoietin/Tie2 system; it promotes VSMC proliferation by upregulating Angiopoietin-2(Ang-2) through PAR2 [173] or lncRNA-LIPCAR signaling [174] while simultaneously inducing endothelial apoptosis by downregulating the Ang-2/Survivin axis, a process antagonized by Angiopoietin-1 to maintain vascular integrity [175,176]. Second, oxLDL significantly dysregulates the Platelet-Derived Growth Factor(PDGF) signaling axis by establishing an autocrine/paracrine loop (e.g., PDGF-AA) that sustains VSMC hyperplasia and neointimal formation, a phenomenon validated in ApoE/LDLR^−/−^ models and implicated in the restenosis of vascular grafts [177,178,179,180]. Third, the FGF family plays a critical mitogenic role; oxLDL and lysophosphatidylcholine induce the release of FGF-1 and FGF-2, driving smooth muscle proliferation and vascular wall thickening in a concentration-dependent manner [181,182,183,184], although this may be accompanied by impaired endothelial repair [185]. Finally, oxLDL utilizes the chemokine network, specifically CXCL16 (SR-PSOX), to facilitate a “chemotaxis-phagocytosis” feedback loop. Regulated by ADAM10-mediated shedding, this receptor promotes oxLDL uptake and foam cell formation, thereby exacerbating lipid deposition and inflammatory angiogenesis [186,187].

It is noteworthy that the regulatory network of oxLDL on macrophage and endothelial cell function exhibits a high degree of complexity. In macrophages, beyond the classic inflammatory pathways, the exchange protein directly activated by cAMP 1(Epac1) signaling pathway has been shown to regulate oxLDL responses and foam cell formation [188]. Concurrently, the body has evolved intricate feedback mechanisms; for example, scavenger receptor class B type I (SR-BI) can inhibit oxPL-induced inflammation [189], while oxPLs themselves can induce the release of CD36 into circulation as a biomarker [69]. In endothelial cells, oxLDL also activates non-canonical pathways, such as the P2Y2 receptor amplification loop [190] or the mechanosensitive ion channel Piezo1 [191]. Furthermore, oxLDL-induced microvesicles containing pro-angiogenic signals (e.g., miR-92a-3p) enable long-range intercellular communication [192]. These emerging discoveries depict a dynamic regulatory landscape, offering new avenues for precise intervention (Figure 4).

In conclusion, the consistent pro-angiogenic effect of oxLDL across diverse diseases is rooted in a conserved molecular program. At its core is the pathological synergy between macrophages and endothelial cells: macrophages are reprogrammed into sources of VEGF, while endothelial cells become the executive units of angiogenesis. However, it is critical to recognize that this pathogenic program operates with significant redundancy. While the “inflammatory activation–oxidative stress–cellular reprogramming” cascade primarily converges on VEGF signaling, oxLDL simultaneously mobilizes VEGF-independent bypass tracks—including the Angiopoietin-2, PDGF, FGF, and CXCL axes. This “multi-channel” strategy constitutes a robust pathogenic network, allowing oxLDL to sustain pathological vascular growth even when the central VEGF highway is compromised. Ultimately, this offers a molecular rationale for the clinical challenge of resistance to anti-VEGF monotherapies, highlighting the need for multi-target therapeutic strategies.

## 6. Advances in Targeting oxLDL for Therapy

As a critical pathogenic factor in multiple neovascular diseases, oxLDL and its downstream lipid peroxidation products have garnered widespread research interest as a potential therapeutic target. In recent years, investigators have explored the possibility of treating these diseases by either inhibiting the formation of oxLDL or blocking its interaction with target cell receptors.

### 6.1. Antioxidant Therapy Targeting LDL Oxidation

Given that the formation of oxLDL and its pro-angiogenic mechanisms are closely dependent on oxidative stress, antioxidants exhibit significant potential for inhibiting oxLDL generation. Various antioxidants, including vitamin C [193] and vitamin E [194], have been demonstrated to inhibit the formation of oxLDL. By reducing systemic oxidative stress, these agents can effectively lower oxLDL levels, thereby attenuating their pro-inflammatory and pro-angiogenic effects. A recent randomized controlled trial showed that supplementation with alpha-lipoic acid reduced the levels of oxLDL-associated Lp-PLA2 in patients with type 2 diabetes [195], suggesting that antioxidant therapy may exert cross-organ protective effects by suppressing oxLDL-related inflammation.

Furthermore, several natural compounds of plant origin, including phenylpropanoids, flavonoids, terpenoids, and alkaloids, have also demonstrated significant antioxidant activity [196,197] and can effectively reduce systemic oxLDL levels [198]. Quercetin, a common flavonoid, is particularly noteworthy for its antioxidant mechanism. It not only directly scavenges free radicals but also enhances the activity of the body’s endogenous antioxidant enzymes. This dual-action mechanism allows it to effectively regulate the balance between oxidation and antioxidation, thereby inhibiting oxidative stress and possessing the capability to suppress oxLDL formation [199]. Notably, quercetin supplementation has been reported to exert significant neuroprotective effects in a rat model of diabetic retinal injury [200]. The antioxidant effects of these natural compounds are primarily achieved through the following mechanism: under conditions of oxidative stress, these antioxidant components can disrupt the inhibition of Nrf2 by Kelch-like ECH-associated protein 1 (Keap1), promoting Nrf2 translocation to the nucleus. There, through transcription factors such as cAMP response element-binding protein (CREB), it activates the Nrf2/heme oxygenase-1 (HO-1) signaling pathway. This, in turn, modulates the expression of downstream antioxidant and phase II detoxifying enzymes, including HO-1, NAD(P)H quinone oxidoreductase 1 (NQO1), superoxide dismutase (SOD), and glutathione peroxidase (GSH-Px) [196]. Concurrently, this pathway facilitates the removal of ROS and other harmful substances, promoting antioxidant, anti-inflammatory, and anti-endothelial cell apoptosis effects [196], as well as inhibiting foam cell formation [201]. However, research by Bahar Kartal and colleagues has revealed that quercetin exhibits differential effects on the angiogenic process under varying dosage conditions; its role is not limited to inhibiting neovascularization but may even promote it under specific circumstances [202]. Currently, pharmacokinetic and clinical research on these natural extracts and monomers remains limited, with few studies on their toxicity and target-organ effects. Nevertheless, these compounds continue to show broad therapeutic promise in antioxidant therapy, particularly in the prevention and management of neovascular diseases and other conditions associated with oxidative stress (Table 1).

### 6.2. Blocking oxLDL Scavenger Receptors

oxLDL exerts its pro-inflammatory and pro-angiogenic effects by binding to multiple scavenger receptors, with the three most prominent being LOX-1, CD36, and SR-A. These receptors play a crucial role in the uptake of oxLDL and the activation of downstream signaling pathways, particularly in neovascular diseases, where they promote the pathological process by mediating foam cell formation and inflammatory responses. Therefore, the development of antagonists targeting these receptors has become an important strategy for treating oxLDL-related diseases. LOX-1 is a specific receptor for oxLDL that is widely expressed on vascular endothelial cells, macrophages, and smooth muscle cells. Its binding with oxLDL activates intracellular pro-inflammatory signaling pathways, promoting neovascularization and the formation of atherosclerotic plaques [167,168]. Currently, LOX-1 inhibitors have demonstrated favorable interventional effects in multiple studies; by inhibiting the interaction between oxLDL and LOX-1, they can effectively suppress oxLDL-induced inflammatory responses and neovascularization. Preliminary studies indicate that these drugs have a significant effect on reducing atherosclerotic plaque formation and intraplaque neovascularization [21,203]. For example, the natural compound curcumin has been shown to significantly downregulate the aberrant high expression of LOX-1 in human umbilical vein endothelial cells (HUVECs) induced by saturated fatty acids (e.g., palmitic acid) by inhibiting endoplasmic reticulum stress. This is accompanied by an alleviation of endothelial cell lipotoxicity, recovery of cell viability, and improvement in vascular function [204]. This suggests that targeted regulation of LOX-1 expression or function is a potential therapeutic strategy for maintaining endothelial cell homeostasis and mitigating vascular pathological damage. Additionally, small molecule inhibitors and monoclonal antibodies against LOX-1 have shown promise in preclinical models for alleviating oxLDL-mediated vascular damage [21,203,205]. Future clinical trials can further validate the safety and efficacy of these drugs, thereby providing new intervention strategies for oxLDL-related diseases.

CD36 and SR-A are two classes of scavenger receptors that play important roles in neovascular diseases through the uptake of oxLDL. Although drug development targeting CD36 and SR-A is still in its preliminary stages, inhibiting their function is considered a potential strategy for preventing oxLDL-related pathologies. Recent studies have found that various compounds can inhibit CD36-mediated oxLDL uptake. For example, sulfo-N-succinimidyl oleate (SSO) has been shown to specifically inhibit CD36 in in vitro experiments, thereby significantly reducing foam cell formation [206]. Furthermore, chitosan oligosaccharides (COS) have been demonstrated to inhibit foam cell formation by downregulating the expression of both CD36 and SR-A in mouse cells treated with oxLDL [207], suggesting their potential application in various neovascular diseases.

Collectively, as the primary receptors for oxLDL, LOX-1, CD36, and SR-A all possess the potential to be therapeutic targets. Among them, the development of LOX-1 inhibitors is the most advanced, with candidate drugs having entered the preclinical evaluation stage [205]. In contrast, the development of drugs targeting CD36 and SR-A is relatively lagging and remains in the preliminary stages. In the future, a multi-target combination intervention strategy may more effectively control the progression of oxLDL-related diseases, offering new therapeutic options for neovascular conditions (Table 1).

### 6.3. Targeting the oxLDL/β2-GPI Complex and Its Ligand oxLig-1

β2-glycoprotein I (β2-GPI) can form complexes with oxLDL, which play a significant role in the development and progression of atherosclerosis [208]. The formation of these complexes is primarily mediated by a specific ligand on the surface of oxLDL—7-ketocholesteryl-9-carboxynonanoate (oxLig-1) [209]. This ligand serves as the key epitope for the interaction between β2-GPI and oxLDL in pathological states such as autoimmune atherosclerosis [208,210]. Research has revealed that the biological activity of oxLig-1 is highly dependent on the ω-carboxyl group in its molecular structure; methylation of this group significantly reduces its binding affinity to β2-GPI and other receptors [11,28,211]. These oxLDL/β2-GPI complexes can promote macrophage uptake of antibody-dependent lipid complexes and accelerate foam cell formation, driving the progression of atherosclerosis [208,212], and this process is also closely linked to neovascularization mechanisms.

Studies have shown that oxLig-1 specifically binds to the fifth domain (Domain V) of β2-GPI, which is the key site mediating the interaction between oxLDL and β2-GPI [212]. Recombinant β2-GPI-DV (rβ2-GPI-DV) can competitively inhibit the binding of oxLig-1 to β2-GPI, significantly inhibiting the formation of the pathogenic complexes [212]. Subsequent research in the serum of patients with antiphospholipid syndrome (APS) further confirmed that rβ2-GPI-DV not only effectively dissociates pre-formed oxLDL/β2-GPI complexes but also prevents the formation of new ones, offering a novel intervention strategy for preventing related vascular complications [211].

Furthermore, the pathophysiological effects of oxLig-1 are pleiotropic. Recent studies have indicated that oxLig-1 is not only a key epitope mediating the binding of oxLDL to the scavenger receptor CD36 [213] but can also act as an endogenous ligand that activates the PPARγ signaling pathway, which in turn upregulates the expression of ABCA1 via LXRα to promote cholesterol efflux [11]. Recent molecular modeling and in vivo and in vitro studies have demonstrated that oxLig-1 can competitively bind to the CD36 receptor with a significantly higher affinity than saturated and monounsaturated long-chain fatty acids. This action inhibits fatty acid-induced lipid accumulation in hepatocytes, showcasing a potential lipid-lowering effect [210]. Therefore, the application of rβ2-GPI-DV to competitively inhibit the formation of oxLDL/β2-GPI complexes has become an important therapeutic avenue for suppressing their pathological effects [212]. More importantly, as our understanding of oxLig-1 as a molecule interacting with multiple receptors deepens, its research value in vascular diseases has become increasingly prominent, making it a significant potential target for the development of novel therapeutic strategies for related diseases (Table 1).

### 6.4. Emerging Therapeutic Strategies

oxLDL can also exert its effects through various other receptors and signaling pathways. Studies have found that Saikosaponin can mitigate oxLDL-induced vascular endothelial cell injury, apoptosis, and inflammatory responses by inhibiting the MAPK signaling pathway [214]. Diosgenin promotes the polarization of macrophages toward an anti-inflammatory phenotype by downregulating the NF-κB signaling pathway [215]. Additionally, researchers are exploring more potential targets, such as ROS generation pathways [216], which play a critical role in oxLDL-induced inflammation and neovascularization. Notably, NF-κB has been shown to have dual functions, capable of mediating both pro-inflammatory responses and participating in anti-inflammatory regulation [217,218]; its specific role varies depending on the pathological context and requires further elucidation.

Concurrently, enhancing the capacity of high-density lipoprotein (HDL) to metabolize and clear oxLDL and its downstream lipid peroxidation products represents an emerging therapeutic strategy. Research indicates that HDL not only acts as an acceptor vehicle for pro-inflammatory molecules (such as oxPC and Lyso-PC), facilitating their transfer from oxLDL, but its own enzymatic machinery (such as Lp-PLA2 and LCAT) can also efficiently hydrolyze or remodel these lipids through esterification, thereby neutralizing their biological activity [30,219]. This dynamic metabolic clearance process not only helps explain why the actual in vivo concentrations of oxLDL and its downstream product Lyso-PC are lower than might be expected but also suggests that enhancing HDL’s lipid metabolic activity could become a novel and effective therapeutic strategy for reducing the overall pathogenicity of oxLDL.

Beyond exploring novel signaling pathways, recent breakthroughs have highlighted the immense potential of immunotherapeutic approaches specifically targeting oxLDL. In the context of atherosclerosis, Schwab et al. developed a pioneering regulatory T cell (Treg) therapy engineered with a chimeric antigen receptor (CAR) specific for oxLDL. This oxLDL-targeted CAR-Treg strategy not only effectively reduced macrophage foam cell formation in vitro but also significantly inhibited atherosclerotic plaque progression in immunocompetent mouse models, offering a precise immunomodulatory tool for vascular inflammation. Furthermore, targeting oxLDL has shown promise in enhancing the efficacy of cancer immunotherapy through combination strategies [220]. Zeng et al. revealed that oxLDL accumulation in the tumor microenvironment metabolically primes CD36-expressing cancer-associated fibroblasts (CAFs), leading to lipid peroxidation-dependent immunosuppression [221]. Crucially, they demonstrated that combining antioxidant vitamin E with anti-PD-1 blockade could diminish this CAF population, thereby overcoming resistance to immunotherapy.

It is important to recognize that the pathological effects of oxLDL are systemic and not confined to specific organs. As a cross-organ biomarker, oxLDL can affect the entire vascular system via blood circulation. A recent study demonstrated that endurance exercise training can significantly reduce aortic oxLDL levels and improve endothelial function in diabetic rats [222]. This research, from the perspective of non-pharmacological intervention, provides complementary evidence to drug-based approaches and highlights the potential of oxLDL as a modifiable target. In the future, more research is anticipated to further explore the regulatory mechanisms of oxLDL in cross-organ diseases to drive the development of innovative therapeutic strategies (Table 1).

**Table 1:** Advances in Targeting oxLDL for Therapy.

Treatment Strategy	Target	Representative Compound/Methods	Refs.
Antioxidant therapy	Reduce ROS/Inhibit oxLDL formation	Vitamin C Vitamin E Alpha lipoic acid Phenylpropanoids Flavonoids Terpenoids Alkaloids Quercetin	[193, 194,195,196,198,199]
Targeting oxLDL Signaling	LOX-1	Curcumin MEDI6570^®^ (AstraZeneca)	[204] [21,205]
	CD36/SR-A	SSO COS	[206] [207]
	oxLDL/β2-GPI complex	rβ2-GPI-DV	[208,212]
	MAPK/NF-κB pathways	Saikosaponin Diosgenin	[214] [215]
Enhancing Metabolic Clearance	HDL function	Targeting Lp- PLA2/LCAT activity	[30,219]
Non-pharmacological Intervention	Systemic oxLDL levels	Endurance exercise	[222]
Immunotherapy & Combination Strategy	oxLDL-driven inflammation	Anti-oxLDL CAR-Tregs	[220]
	CD36^+^ CAFs/Lipid peroxidation	Vitamin E + Anti-PD-1 antibody	[221]

This table summarizes current and emerging therapeutic strategies aimed at inhibiting oxLDL formation, blocking its signaling pathways, or enhancing its metabolic clearance, along with representative compounds and intervention methods.

## 7. Conclusions and Perspectives

This review synthesizes current evidence elucidating the pivotal role of oxLDL as both a cross-organ biomarker and a key driver in multiple neovascular diseases. The available evidence indicates that oxLDL and its downstream lipid peroxidation products drive pathological angiogenesis in different tissue microenvironments, a mechanism that integrates disease-specific cellular regulatory pathways with a highly conserved set of common molecular mechanisms. The pathological process is initiated by the abnormal accumulation of oxLDL in specific tissue microenvironments (the ocular fundus, arterial wall, and tumors), which then triggers disease onset through interactions with specific cell types (e.g., RPE cells, VSMCs, and tumor cells). Subsequently, this process activates a universal molecular program in macrophages and endothelial cells centered on an “inflammation–oxidative stress–VEGF upregulation” axis, which ultimately drives neovascularization. This duality not only reveals the intrinsic molecular link between neovascular ophthalmopathy, atherosclerosis, and tumors but also consolidates the theoretical basis for oxLDL as a therapeutic target with high clinical translational potential across these diseases.

These common and specific mechanisms converge on a central concept: oxLDL is a key “cross-organ” mediator that links systemic lipid dysregulation to local, pathological neovascularization. The ‘cross-organ’ nature of oxLDL does not imply interspecies homology, but rather highlights the high conservation of the oxLDL-mediated signaling axis across diverse organ microenvironments. It reflects the ability of oxLDL to activate a universal pro-angiogenic molecular program across distinct diseases, while the final pathological phenotype is determined by its interaction with the specific tissue microenvironment and cell types involved. This duality further substantiates the central role of oxLDL in multisystem vascular pathologies.

However, the regulation of the oxLDL signaling network is not a simple linear relationship; its pleiotropy and dependence on the pathological microenvironment pose key challenges to a full understanding of its pathological role. oxLDL and its downstream lipid peroxidation products can exhibit contradictory biological effects under different conditions. In most cases, oxLDL is pro-angiogenic, but some studies have revealed an inhibitory effect under specific circumstances. For instance, one study found that in a scratch wound healing assay, oxLDL significantly reduced the migratory capacity of human microvascular endothelial cells (HMEC-1) [223]. A recent study found that oxLDL can induce the expression of fatty acid-binding protein 3 (FABP3) in human coronary artery endothelial cells, and FABP3, in turn, impairs their function and suppresses neovascularization by activating the ERK/p38/STAT1 signaling pathway [224]. Furthermore, the effects of oxLDL’s downstream lipid peroxidation products are similarly complex and can be concentration-dependent. For example, in vitro experiments have shown that 4-HNE promotes tube formation by endothelial cells at low concentrations (0.5–1 μM) but inhibits this process at high concentrations [225]. Similarly, Lyso-PC has been found in some studies to directly inhibit the pro-angiogenic functions of endothelial progenitor cells (EPCs) [226], or its accumulation has been negatively correlated with angiogenic capacity under certain molecular perturbations [227]. These seemingly contradictory phenomena underscore the complexity of the oxLDL signaling network, presenting significant challenges for therapeutic targeting and warranting further elucidation of the underlying mechanisms.

Furthermore, elucidating how oxLDL signaling pathways integrate or engage in crosstalk with other unique pathological factors within each tissue is of paramount importance. Prioritizing the elucidation of the specific cellular interaction profiles and signal network differences of oxLDL in various pathological microenvironments will provide a basis for understanding disease heterogeneity and developing personalized therapeutic regimens. Ultimately, the goal of this field is to advance more precise and safer targeted therapeutic strategies into the clinic. Future drug development should transcend single-target approaches to explore multi-target synergistic interventions or allosteric modulators that selectively block specific pathological signaling branches. At the same time, emerging areas, such as long-range signaling mediated by microvesicles/exosomes and the role of newly discovered pathways like Epac1 in the cross-organ effects of oxLDL, offer new directions for innovative therapeutic strategies.

## Data Availability

No new data were created or analyzed in this study. Data sharing is not applicable to this article.

## Abbreviations

The following abbreviations are used in this manuscript:

4-HNE	4-hydroxynonenal
7-KC	7-ketocholesterol
ATX	Autotaxin
β2-GPI	β2-glycoprotein I
CD36	CD36 (a class B scavenger receptor)
CNV	Choroidal neovascularization
DAMPs	Damage-associated molecular patterns
EndMT	Endothelial-Mesenchymal Transition
EMT	Epithelial–mesenchymal transition
Epac1	Exchange protein directly activated by cAMP 1
FC	Free cholesterol
HDL	High-density lipoprotein
HIF-1α	Hypoxia-inducible factor 1-alpha
HO-1	Heme oxygenase-1
ICAM-1	Intercellular Adhesion Molecule-1
LDL	Low-density lipoprotein
LOOH	Lipid hydroperoxides
LOX-1	Lectin-like oxidized low-density lipoprotein receptor-1
Lp-PLA2	Lipoprotein-associated phospholipase A2
LPA	Lysophosphatidic acid
Lyso-PC	Lysophosphatidylcholine
MAPK	Mitogen-activated protein kinase
MMPs	Matrix metalloproteinases
MCP-1	Monocyte Chemoattractant Protein-1
nAMD	Neovascular age-related macular degeneration
NF-κB	Nuclear factor kappa-B
NOX	NADPH oxidase
Nrf2	Nuclear factor erythroid 2-related factor 2
oxLig-1	7-ketocholesteryl-9-carboxynonanoate
oxLDL	Oxidized low-density lipoprotein
oxPC	Oxidized phosphatidylcholines
oxPL	Oxidized phospholipids
PC	Phosphatidylcholine
PDR	Proliferative diabetic retinopathy
PI3K/Akt	Phosphoinositide 3-kinase/protein kinase B
PPARγ	Peroxisome proliferator-activated receptor gamma
PUFAs	Polyunsaturated fatty acids
rβ2-GPI-DV	Recombinant β2-GPI-Domain V
ROS	Reactive oxygen species
RPE	Retinal pigment epithelium
SASP	Senescence-associated secretory phenotype
SR-A	Scavenger Receptor class A
SSO	Sulfo-N-succinimidyl oleate
TAMs	Tumor-associated macrophages
TLRs	Toll-like Receptors
TME	Tumor microenvironment
VCAM-1	Vascular Cell Adhesion Molecule-1
VEGF	Vascular endothelial growth factor
VM	Vascular mimicry
VSMCs	Vascular smooth muscle cells
